# Passiflora extract for the management of cannabinoid withdrawal syndrome and dependence: evidence from a case report of an adolescent girl with bipolar disorder

**DOI:** 10.1192/j.eurpsy.2026.10849

**Published:** 2026-07-31

**Authors:** M. M. Mensi

**Affiliations:** 1 Brain and Behavioral Sciences, University of Pavia; 2 Child and Adolescent Psychiatry, IRCCS Mondino Foundation, Pavia, Italy

## Abstract

**Introduction:**

Given the rising potency of cannabis, the diversification of consumption methods, and the decreasing perceived risk, cannabis represents a particularly critical substance to monitor during childhood. Adolescent cannabis use is associated with chronic cognitive, psychosocial, psychiatric, and physical worst outcomes. There is currently no pharmacologic treatment for cannabis withdrawal. Among the symptoms of cannabis withdrawal, patients tend to perceive anxiety most strongly, partly due to the belief that cannabis helps especially with this emotion.

**Objectives:**

It is therefore important to find support strategies to manage withdrawal symptoms and encourage cessation of use. This study aims to evaluate the efficacy of a new drug, containing as active principle a dry extract of Passiflora Incarnata L., Herba (200 mg tabs equivalent to 700 – 1000 mg of Passiflora) in managing cannabinoid withdrawal symptoms and subsequent cannabinoid disengagement.

**Methods:**

A 16-year-old patient with suicidal ideation and bipolar disorder. Toxicological screening revealed cannabinoids (96 ng/ml). The girl reported smoking cannabis 6 times a day. She justified this use as a form of acute treatment for her perceived anxiety. Upon discontinuation, she began to experience : intense anxiety leading to self-harm, depression, irritability, and insomnia. These symptoms were treated with:

1. Delorazepam drops: 26 drops at 10 p.m. + fast-acting melatonin 1 mg + slow-release melatonin 1 mg for insomnia;
2. Passiflora incarnata 2 tablets: 9 a.m.-9 p.m. for anxiety;
3. Passiflora incarnata 1 tablet as needed: up to 6 tablets/day if psychomotor agitation.

**Results:**

The patient was able to tolerate the withdrawal symptoms: her insomnia resolved on the 2nd day, and her anxiety symptoms decreased after 7days, even though the concentration of cannabinoids in her urine was still positive (56 ng/ml). The possibility of taking up to 6 Passiflora incarnata tablets per day, according to the patient’s perceived need, allowed her to maintain a sense of self-care by replacing each smoke with a tablet in an exactly equivalent manner. After discharge, the patient tested negative for cannabinoids in a toxicology test one month later.

**Conclusions:**

Passiflora incarnata is a promising option for treating withdrawal symptoms and cannabinoid dependence in adolescents. This molecule is useful for treating anxiety, which is one of the most common reasons for cannabis use in this age group. Furthermore, given the low number of side effects, young people can manage their own administration, maintaining a feeling of self-care similar to that of smoking. Finally, this molecule allows the use of benzodiazepines to be minimised, reducing the risk of tolerance and dependence associated with these molecules.

**References:**

Ansseau2004;Carminati2024;Crespi2024;Hashemi2023;Zanardi2023.

**Disclosure of Interest:**

None Declared

